# Advance in the Thermoinhibition of Lettuce (*Lactuca sativa* L.) Seed Germination

**DOI:** 10.3390/plants13152051

**Published:** 2024-07-25

**Authors:** Jinpeng Wei, Qi Zhang, Yixin Zhang, Le Yang, Zhaoqi Zeng, Yuliang Zhou, Bingxian Chen

**Affiliations:** 1 Guangdong Provincial Key Laboratory for Crop Germplasm Resources Preservation and Utilization, Agro-Biological Gene Research Center, Guangdong Academy of Agricultural Sciences, Guangzhou 510640, China; 2 College of Agriculture, South China Agricultural University, Guangzhou 510642, China; 3 College of Agriculture and Biology, Zhongkai University of Agriculture and Engineering, Guangzhou 510550, China

**Keywords:** lettuce seeds, high-temperature germination, thermoinhibition, hormones, interactions, genetic expression

## Abstract

Thermoinhibition refers to the inability of seeds to germinate when inhibited by high temperatures, but when environmental conditions return to normal, the seeds are able to germinate rapidly again, which is different from thermodormancy. Meanwhile, with global warming, the effect of the thermoinhibition phenomenon on the yield and quality of crops in agricultural production is becoming common. Lettuce, as a horticultural crop sensitive to high temperature, is particularly susceptible to the effects of thermoinhibition, resulting in yield reduction. Therefore, it is crucial to elucidate the intrinsic mechanism of action of thermoinhibition in lettuce seeds. This review mainly outlines several factors affecting thermoinhibition of lettuce seed germination, including endosperm hardening, alteration of endogenous or exogenous phytohormone concentrations, action of photosensitizing pigments, production and inhibition of metabolites, maternal effects, genetic expression, and other physical and chemical factors. Finally, we also discuss the challenges and potential of lettuce seed germination thermoinhibition research. The purpose of this study is to provide theoretical support for future research on lettuce seed germination thermoinhibition, and with the aim of revealing the mechanisms and effects behind lettuce seed thermoinhibition. This will enable the identification of more methods to alleviate seed thermoinhibition or the development of superior heat-tolerant lettuce seeds.

## 1. Introduction

The phenomenon of global warming has emerged as a significant environmental concern in the context of the intensification of the greenhouse effect. It is anticipated that future increases in temperature will have a detrimental impact on all living organisms and ecosystems on Earth, with varying degrees of impact observed [1]. The aforementioned issues have led to the prioritization of research on seed growth and development at elevated temperatures [1,2,3]. Seeds are the most crucial production materials in the agricultural production process, and their germination quality directly affects the success or failure of agricultural production. Seed germination refers to the process of viable dry seeds absorbing water until the radicle emerges from the seed coverings. This process involves numerous physiological, biochemical, and molecular biological changes that cannot be directly observed, as well as the morphological characteristic of the radicle emerging from the seed coverings, which is the only visible sign [4,5,6]. According to the definition, the process after the radicle emerges from the seed coverings belongs to the post-germination and seedling growth stages. Then, the impact of temperature on seed germination is multifaceted. For non-dormant seeds, the germination rate (which is inversely related to the time taken for germination after imbibition) typically increases from the minimum or base temperature (Tb) up to the optimum temperature (To), and then gradually decreases from the optimum temperature to the maximum temperature (Tc), until germination ceases [7]. In other words, before reaching the highest germination temperature, the germination rate increases with rising temperature, which is generally consistent with the thermodynamic principles that govern the effects of external temperature on plant growth. Among these, the problems of seed “thermoinhibition” and “thermodormancy” are the most commonly encountered phenomena by seeds germinating at high temperatures. Thermoinhibition refers to the phenomenon whereby seed germination is inhibited following immersion at high temperatures. However, upon restoration of the inhibiting factors, such as temperature, humidity, and light, to the normal state of seed germination, the seed will germinate rapidly. Consequently, thermoinhibition is a reversible phenomenon [6]. In contrast, thermodormancy is typically a secondary dormancy phenomenon that arises in hydrated seeds as a consequence of prolonged exposure to high temperatures that are unsuitable for germination. Due to its irreversibility, even if external conditions conducive to seed germination are provided at a later stage, the seeds do not germinate rapidly; only after application of special treatment can seeds resume germination [8,9]. Among these, seed thermoinhibition is a widespread phenomenon in many winter annual or biennial species, which prevents seeds from germinating normally during the water-deficient summer. However, the complete mechanism of seed thermoinhibition is not yet fully understood. It is known that after-ripening and cold stratification can alleviate seed dormancy, but it is necessary to study whether after-ripening and cold stratification can reduce seed thermoinhibition.

Although there is a lot of research on the effects of temperature on seed germination and dormancy in a variety of crops [7]. Scientists are still in the early stages of understanding the genetic and physiological mechanisms by which seeds perceive external temperature signals and regulate germination and dormancy [10]. So, this paper presents a comprehensive overview of the research conducted so far on the thermoinhibition of lettuce seed germination. Lettuce (*Lactuca sativa* L.) is an annual or biennial herb of the genus Lettuce in the Asteraceae family, also known as lettuce. It is native to the present-day Mediterranean area. During the extensive domestication process of lettuce, thermoinhibition has been preserved. This is why the germination of most commercial lettuce seeds is significantly affected by high temperatures, with the specific temperature depending on the heat tolerance of the variety and the seed production environment. However, due to the presence of thermoinhibition, this directly leads to modern lettuce varieties entering dormancy when exposed to high temperatures during sowing. This issue is particularly severe in winter lettuce-growing areas. Generally, the optimal germination temperature for most lettuce varieties is between 15 °C and 22 °C [11]. Consequently, lettuce is regarded as a species particularly susceptible to high temperatures, with seeds being particularly sensitive to heat [12]. Currently, to obtain lettuce seeds that can germinate normally at temperatures far above 22 °C, an expensive “priming” treatment is required to stimulate the seeds. Seed priming allows for controlled hydration of the seeds, enabling them to complete the first steps of the germination process before being dried back to their original moisture content and stored until planting, thereby alleviating thermoinhibition by artificially increasing the maximum temperature at which germination occurs. However, this process is not only time-consuming and labor-intensive but also poses issues such as “reducing the shelf life of seeds” and “over-priming causing damage to seed root tissues”. Therefore, developing lettuce varieties that can germinate at high temperatures without the need for priming is the current mainstream direction. The key prerequisite for this direction is to elucidate the physiological mechanisms and genetic effects of thermoinhibition in lettuce seeds. Consequently, numerous scientists have conducted research into the growth of lettuce under heat-stress conditions. For example, in response to external stress, lettuce exhibits significant morphological changes, including an increase in stem node height, a reduction in leaf thickness, and the development of septic lesions. Concurrently, a decline in nutrient content occurs, which affects commercial yields and results in economic losses [13,14,15].

For seeds of lettuce, both temporary germination inhibition (thermoinhibition) and permanent germination inhibition (heat stress) are caused by the hardening of the endosperm, which prevents the normal protrusion of the radicle and thus blocks seed germination [16]. Moreover, thermoinhibition is naturally reversible when the normal germination environment is restored. In contrast, heat dormancy cannot be restored naturally once it has been implanted in the seed. Consequently, in the case of heat-dormant lettuce seeds, the seeds require some kind of treatment to germinate even if environmental conditions favorable for germination exist [12]. After the high-temperature stress is alleviated, certain varieties of lettuce may naturally enter a state of primary dormancy before harvest. However, this dormancy is automatically lifted following storage for a specified period, and the mechanism varies depending on the lettuce genotype [16]. The identification of lettuce genotypes tolerant to high temperatures has an effect at 260 days after harvest. Consequently, when utilizing lettuce seeds, it is vital to meticulously examine the germination of the variety as documented in the literature to ascertain whether it is heat-dormant or heat-suppressed [17]. 

However, the intricate interplay of various factors renders the investigation of the underlying mechanisms and physiology of lettuce seed germination in the context of thermoinhibition a complex and challenging endeavor. It is noteworthy that the effects of different factors on the thermoinhibition of lettuce seed germination vary in lettuce; in other words, different genotypes of lettuce exhibit varying coping strategies in response to high temperatures. Consequently, the conclusions drawn from a study may vary among different varieties of lettuce. At present, the results of studies on the inhibition of lettuce seed germination by heat are still relatively abundant. For instance, in the case of Grand Rapids seeds, the seed endosperm and seed coat can act as a restriction on the extension of the radicle [18], while changes in the concentration of agonists or phytohormones such as GA, ABA, ETH [19,20], the role of photosensitive pigments in seeds [21], and the expression of genes related to temperature changes can affect seed germination at high temperatures. Given the complex interactions among factors affecting seed germination under high temperatures, and considering that the exploration of these interactions is still in its early stages, this paper aims to present the current research findings on heat suppression in lettuce seeds to researchers engaged in or preparing to conduct related experiments in a concise and understandable manner. Therefore, due to the limited space available, this paper will categorize and discuss each factor that influences heat suppression in lettuce seeds separately.

## 2. Effects of Endosperm

**Figure 1:**
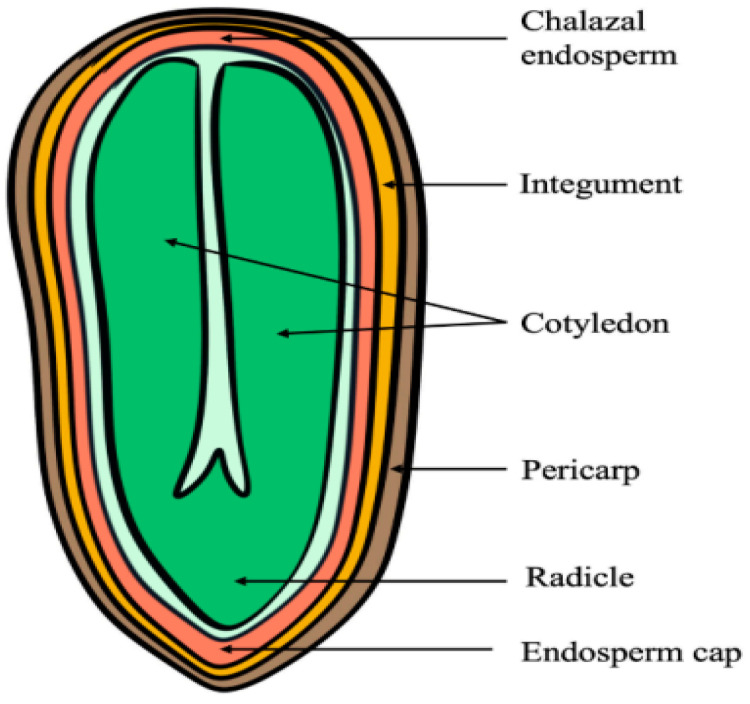
Schematic diagram of lettuce seed profile. Adapted from [37].

For the majority of species, seed germination is contingent upon the participation of the embryo. For instance, [22] it has been experimentally demonstrated that certain mechanisms are responsible for seed thermoinhibition, including developmental arrest of the embryo, subsequent protrusion of the radicle, and gradual hardening of the endosperm. In the high-temperature state, embryo coverings are an important factor in lettuce seed germination under the influence of thermoinhibition [18]. The role of different tissues in the production of thermoinhibition varies between different varieties of lettuce seeds. For instance, the germination of Cobham Green lettuce seeds is intimately linked to the pericarp, and the application of hypochlorite at 35 °C can effectively mitigate thermoinhibition in Cobham Green lettuce seeds. However, the degree of impact is not uniform across Empire and Benita lettuce seeds [18]. Nevertheless, the impact of pericarp treatment on heat suppression in Grand Rapids seeds was less pronounced than that of endosperm weakening [23]. In seeds, the endosperm serves a multifaceted role. It provides nutrients and protection to the embryo and also acts as a physical barrier that controls the growth and development of the embryo [24,25]. The endosperm surrounding the embryo is essential for exerting thermoinhibition on lettuce seeds. Furthermore, the rapidity and extent of endosperm attenuation near the apical bead pore of the radicle may be associated with the initiation of thermoinhibition. Meanwhile, the state of the endosperm varies considerably between different varieties of lettuce seeds (Figure 1) [26]. Otherwise, the authors of [27] posit that the greater the heat sensitivity of lettuce varieties, the harder the seed endosperm. Conversely, the softer the endosperm of more heat-tolerant seeds. This is consistent with the study’s findings, which indicate that the endosperm functions as a physical barrier for the embryo, the harder the endosperm tissue, the stronger the barrier, which impedes the normal growth of the embryo and seed germination. The above results indicate that the endosperm is the tissue that most significantly affects the heat resistance of seeds. The end of lettuce seed germination is marked by the successful penetration of the radicle into the endosperm at the end of the bead pore and its protrusion. Furthermore, the mechanical strength of the endosperm of heat-tolerant lettuce seeds was lower than that of seeds of heat-sensitive varieties in a puncture test on the endosperm layer (Figure 1) [27].

To investigate the influence of the endosperm, researchers have designed a number of related experiments. The germination of three types of embryos was observed at 34 °C: complete seed, no seed coat but have endosperm, and no seed coat and endosperm. It was found that the embryo with both the seed coat and endosperm removed was able to germinate normally, while the embryos of the other two cases failed to germinate normally, indicating that the endosperm and the seed coat are thermally inhibitory to the seed [28]. Furthermore, the authors of [29] showed that thermoinhibition of Arabidopsis seed germination is not controlled independently by the embryo, but is achieved by the endosperm. Endosperm phyB can sense high temperatures, which accelerates its transition from the signal-active Pfr form to the inactive Pr form, this process leads to a thermoinhibitory process mediated by phytochrome-interacting factors (PIFs), including PIF1, PIF3, and PIF5. Among them, PIF3 in the endosperm represses the expression of the endosperm ABA catabolic gene *CYP707A1*, which promotes endosperm ABA accumulation and release into the embryo, while at the same time, inducing PIF3 in the endosperm can inhibit the expression of endosperm ABA catabolic gene *CYP707A1*, which promotes the accumulation and release of endosperm ABA into the embryo. At the same time, this inhibits the expression of PIF3 in the embryo, inhibiting the growth and development of the embryo. Thus, it is essential to further explore whether a similar mechanism exists in the endosperm of lettuce seeds to control thermoinhibition.

In addition, the weakening of the endosperm in seeds is caused by relevant autolytic enzymes in their cell walls, which is equally important for lettuce seed germination. Given that enzymes are proteins, this process is affected by temperature and acidity [30]. Endo-β-mannanase (an enzyme that binds to the cell wall) is a crucial enzyme in the weakening of the endosperm layer prior to the emergence of the radicle, which is a pivotal factor in the successful germination of seeds at high temperatures [31]. The enzyme endo-β-mannanase, which is capable of degrading the mannan component of the plant cell wall, is encoded by *LsMAN1*. This enzyme participates in the remodeling and degradation of the plant cell wall. *LsMAN1* is induced by gibberellic acid (GA) and inhibited by abscisic acid (ABA) [32,33]. The mRNA abundance of *LsMAN1* was high in both high-temperature-sensitive and high-temperature-tolerant genotypes of lettuce varieties immediately after radicle emergence at 20 °C. However, the expression was low in seeds that were subjected to heat stress at 35 °C (Figure 1) [34]. Similarly, heat-tolerant species produce more endo-β-mannanase, which weakens endosperm cell wall stiffness and promotes embryo growth and seed germination. Conversely, heat-sensitive seeds with a higher content of mannose and with lower endo-β-mannosidase expression resulted in more difficult seed germination. [35]. By increasing the activity of endo-β-mannanase in lettuce seeds through soaking, the temperature-sensitive variety “Dark Green Boston” achieved 100% germination at 35 °C, whereas unsoaked seeds exhibited a germination rate of only 4% at 35 °C [36]. During the germination of lettuce seeds at room temperature, cell wall hydrolases such as cellulase [37], pectin methylesterase [38], xyloglucan endotransglycosylase [39], and arabinogalactanase [40], and non-enzymatic factors such as reactive oxygen species [41] are involved in the breakdown of the cell wall and the elongation of the radicle, facilitating the penetration of the radicle into the endosperm, these factors relax the cell wall and facilitate the breakthrough of the radicle into the bead pore endosperm. Given that the primary research conclusions regarding various enzymes related to the endosperm cell wall have been conducted under room temperature conditions, and that much of this research is still in its preliminary stages without fully exploring the underlying genetic mechanisms of these functional enzymes, it is reasonable to hypothesize that under high temperatures, these key cell wall hydrolases may either function similarly or be influenced by other factors. Additionally, whether there are certain interrelationships among various hydrolases remains an area of research that is currently lacking.

## 3. Abscisic Acid and Gibberellin

**Figure 2:**
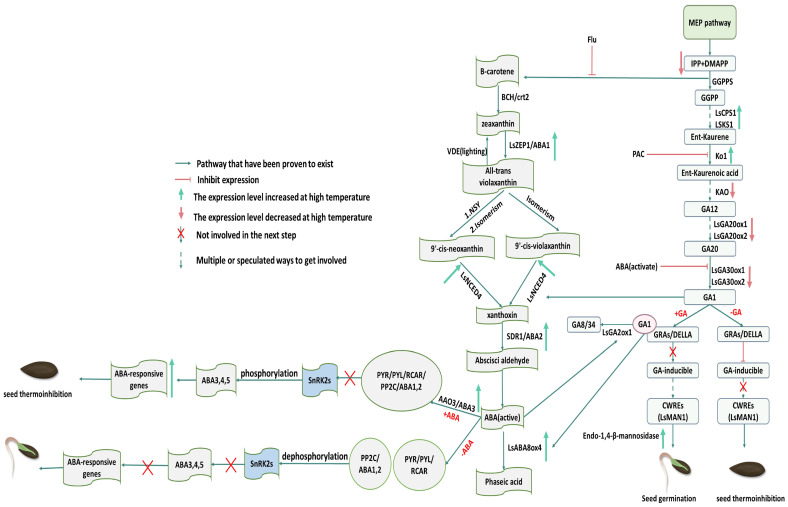
Biosynthesis and signaling pathways of abscisic acid ABA and GA. Adapted from [63,64]. Some of the gene/enzyme names are given in Table 1.

Abscisic acid, a common seed germination inhibitory hormone, is another important element in lettuce seed germination under the influence of thermoinhibition. The two-step epoxidation reaction of *ABA1/LsZEP1*, which catalyzes the conversion of zeaxanthin to violaxanthin, represents the initial critical step in the biosynthesis of abscisic acid (ABA) in lettuce seeds. This process is described in detail in reference [42]. It has been demonstrated that mutation of *LsABA1* results in a reduction in the endogenous ABA content, whereas a single base mutation of *ABA1/LsZEP1* results in a heat-tolerant phenotype in germinating seeds of TG01 and TG10. This suggests that *ABA1/LsZEP1* is possibly a gene of considerable importance in the regulation of heat suppression in lettuce seed germination (Figure 2) [43]. The results of numerous experiments demonstrate that the application of exogenous ethylene, GA, and red light can effectively increase the upper temperature limit of lettuce seed germination, whereas ABA decreases the upper temperature limit of seed germination [44,45,46]. One study [47] proved that the addition of exogenous GA enhanced the oxidative catabolism of ABA in lettuce seeds, resulting in a significantly higher total amount of catabolite metabolites than in lettuce seeds without exogenous GA. The application of these exogenous hormones alone can have some effect on seed germination. Furthermore, the authors of [48] indicated that the maximum temperature of lettuce seed germination mainly depends on the antagonistic effect between GA and abscisic acid. This means that ABA can antagonize the promotional effect of GA and ABA and GA affect the metabolism of other hormones mutually [7]. It has been illustrated that the sensitivity of lettuce seeds to the inhibition of abscisic acid (ABA) is enhanced at high temperatures, resulting in an increase in ABA content, which inhibits gibberellin (GA) synthesis and signaling processes. For example, the sensitivity of lettuce seeds to ABA was found to increase with rising temperatures. For instance, 10 mM ABA inhibits lettuce seed germination at 30 °C, whereas 100 mM ABA does not completely inhibit it at 20 °C [49,50]. Another study indicated that in lettuce seeds, the temperature of thermoinhibition reduces the accumulation and gene expression of certain proteins in the mevalonate pathway and affects the synthesis of the major product isoprenyl pyrophosphate. Since isoprenyl pyrophosphate is a common precursor for GA and abscisic acid synthesis, this effect may amplify the suppressive effect of abscisic acid on lettuce seed germination. (Figure 2) [51]. In general, abscisic acid is highly expressed when lettuce seed germination is heat-inhibited, whereas GA and ethylene-related genes are highly expressed when germination conditions are favorable. The regulation of heat-inhibited germination of lettuce seeds by these three hormones is very tightly linked, and changes in the external ambient temperature constantly affect the operation of this regulatory network [52,53]. Therefore, the current research direction is not limited to the role of a single plant hormone under abiotic stress in plant seeds but rather focuses on the interactions between multiple hormones. ABA, as a relatively well-studied endogenous plant hormone, has been extensively researched for its effects on various stresses in other model crops and plants. This provides a reference for future research on the thermoinhibition of lettuce seeds.

Gibberellin (GA) has a significant promotional effect on seed germination and is able to overcome the germination inhibitory effect induced by abscisic acid (ABA) [54]. In lettuce seeds, the use of GA biosynthesis inhibitors results in the inhibition of seed germination recovery at high temperatures [55], while exogenous GA3 promotes germination by reducing the endogenous ABA content [56]. It is evident that GA may influence seed response to temperature by affecting the early biosynthetic and metabolic pathways of abscisic acid (ABA), and under high temperatures, the biosynthesis and metabolism of GA itself are also impacted. For instance, as homologous genes in lettuce to those in Arabidopsis, three genes of the early GA biosynthesis pathway, *LsCPS1*, *LsKS1*, and *KO1*, exhibit a strikingly similar expression pattern to their homologs in Arabidopsis. This is evidenced by the observation that the expression of these genes is significantly elevated in suckered seeds exposed to light at 35 °C relative to those exposed to light at 20 °C (Figure 2) [57]. In lettuce seeds that had not undergone any treatment, the expression of GA and ethylene biosynthesis-related genes *LsGA3ox1* and *LsACS1* was suppressed following high-temperature imbibition. However, after accelerating germination, the expression of ETH and GA biosynthesis-related genes, *LsACS1* and *LsGA3ox1*, was suppressed after high-temperature seed imbibition, although *LsNCED4* was suppressed, and this mechanism was not reversed by high-temperature imbibition [58]. *LsNCED4* is expressed at the later stages of seed maturation, and its regulatory outcome may be related to the increased expression of GA and ethylene biosynthesis genes; additionally, the *NCED9*-like gene in Arabidopsis and homologs of this gene in other species are important for the conduct of abscisic acid biosynthesis under high-temperature imbibition [4]. It is worth mentioning that a number of studies have demonstrated that *LsGA20ox1* and *LsGA20ox2*, which encode GA20-oxidase, the penultimate step in GA biosynthesis, and *LsGA3ox1*, which encodes gibberellin (GA) 3β-hydroxylase, catalyze the final step in GA1 biosynthesis [59,60,61]. Furthermore, RNAi-*NCED4* lettuce seeds exhibited an upregulation of GA biosynthesis genes *GA3ox1* and *GA20ox1* and a downregulation of *GA2ox1*, encoding a GA-degrading enzyme, at 35 °C. This pattern was found to be essentially similar to that observed in *ABI5*, *ABI3*, and *SNF4*. It was speculated that this phenomenon might be due to a lower content of ABA (Figure 2) [51]. In lettuce, some genes associated with early GA synthesis are upregulated under high-temperature conditions, and it was hypothesized that this could be a feedback inhibition formed by GA with these genes, causing them to be upregulated when reactive gas synthesis is inhibited [49]. It is curious to note that the application of GA alone had a positive effect on alleviating heat suppression in lettuce seeds. In contrast, through the use of fluridone alone, an inhibitor of ABA biosynthesis that blocks phytoene desaturase in the carotenoid synthesis pathway [62], heat suppression in lettuce seeds was prevented at 28 °C. However, at 33 °C, seed sensitivity to ABA increases, and fluridone alone is no longer effective. At this time, the combination of fluridone with GA was able to germinate lettuce seeds normally [48]. This may be attributed to the fact that at 33 °C, fluridone inhibited the biosynthesis of ABA, while exogenous GA3 could promote the decomposition of ABA, reducing the content of endogenous ABA from both synthesis and decomposition. Consequently, the germination threshold ABA content at this time was lower than the germination content of lettuce seeds that were thermally inhibited at 28 °C. Furthermore, the germination of lettuce UC96US26 seeds was found to be inhibited following the use of polythymol (PTF), a GA synthesis inhibitor [6]. The above results indicate that, in addition to controlling ABA levels to ensure they do not exceed the inhibitory threshold, the involvement of GA is also a crucial aspect in alleviating the thermoinhibition of lettuce seeds. Interestingly, many studies have employed artificial or biosynthetic plant hormone inhibitors to demonstrate the role of these hormones, and explaining a phenomenon from multiple perspectives is far more convincing than studying it from a single angle.

Eventually, given the scatter of research investigating the thermoinhibition of lettuce seed germination, there is a pressing need to identify and elucidate the functions of numerous related genes. Table 1 summarizes some genes known or predictive to affect the thermoinhibition of Lettuce seeds. 

## 4. Cytokinin and Ethylene

**Figure 3:**
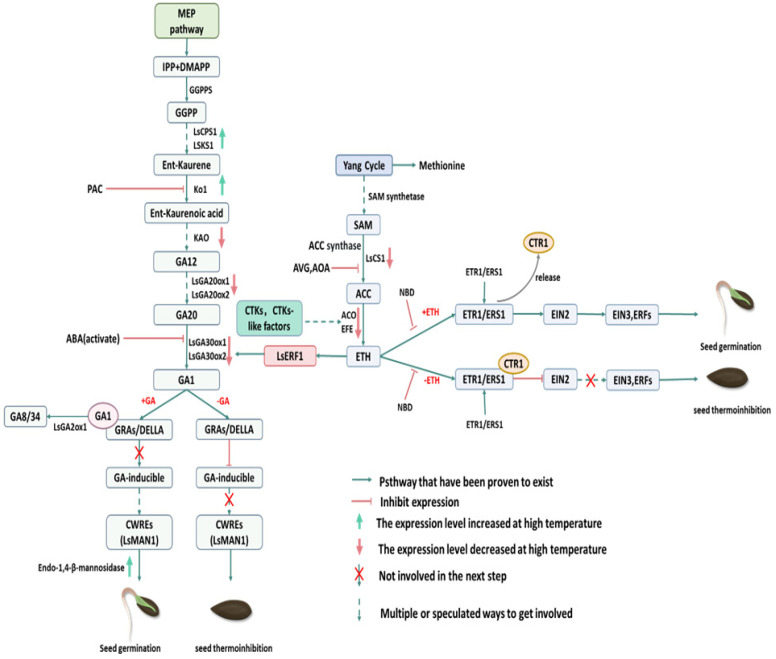
Pathways of ETH and GA biosynthesis and signaling. Adapted from [7,49]. Some of the gene/enzyme names are given in Table 1.

Cytokinin is an endogenous hormone that plays a role in promoting cytoplasmic division, multiple tissue differentiation, and growth. Previous studies have demonstrated that cytokinins can mitigate the thermoinhibition of lettuce seeds at elevated temperatures [87,88]. The content and nature of endogenous cytokinins in lettuce seeds undergo a transformation following the disruption of dormancy through the application of red light irradiation [89]. This indicates that photosensitive pigments facilitate seed germination by regulating the dynamics of endogenous cytokinins in lettuce seeds [90]. These findings elucidate that increasing cytokinin levels may have a positive effect on alleviating thermoinhibition in lettuce seeds. Likewise, the authors of [91] demonstrated that the application of cytokinin resulted in the activation of ethylene-forming enzyme (EFE) or ACC oxidase in lettuce seeds, which enhanced the utilization of ACC. Therefore, we can assume that cytokinins play a pivotal role in regulating ethylene synthesis and germination of lettuce seeds at elevated temperatures (Figure 3).

The sensitivity and tolerance of different lettuce varieties to high temperatures are relative and depend mainly on the genetic characterization of seeds for thermoinhibition, as well as on environmental changes during seed maturation and development. Ethylene, as one of the five plant hormones, is regarded as a pivotal regulator of lettuce germination, particularly in the context of diverse stress environments [92]. It has been demonstrated that seeds matured at elevated temperatures contribute to enhanced lettuce germination at elevated temperatures, which may be associated with elevated ethylene biosynthesis [93]. Conversely, the silencing of *LsNCED4* in Salinas seeds (heat-sensitive lettuce) led to an increase in the transcript levels of two ethylene biosynthesis genes, *ACS1* and *ACO2*, which were also highly transcribed in UC96US26 seeds (heat-resistant lettuce) that had been absorbed at high temperatures [49]. 

Furthermore, it has been demonstrated that ethylene binding to GA and cytokinin has a beneficial effect on the germination of lettuce seeds under thermoinhibition [94]. As the ethylene content of seeds germinating at temperatures below the optimal range decreases, it is important to make sure that ethylene levels are in the normal range [95]. A multitude of studies have identified ethylene synthesizing genes or precursors associated with seed germination under heat-inhibited states of seeds. For instance, as described above, the expression of *ACS1* and *ACO2*, two genes associated with ethylene synthesis, was closely correlated with the thermoinhibition of seed germination in lettuce (Figure 3) [6,49].

The use of exogenous ethylene or its biosynthetic precursor—ACC—is effective in reducing heat stress in the seeds of lettuce and other crops [46,96]. In contrast, AVG (an inhibitor of ethylene biosynthesis) and NBD (an inhibitor of ethylene action) have been shown to block the promotion of thermoinhibition by red light in lettuce seeds under darkness (Figure 3) [45]. This suggests that endogenous ethylene has a mitigating effect not only on the thermoinhibition of lettuce seeds under normal conditions but also on the thermoinhibition of dark environments produced by light-induced lettuce seeds. Additionally, it was observed that exogenous ethylene enhanced endo-β-mannanase activity (participating in endosperm cell wall biodegradation) in heat-sensitive lettuce seeds at 35 °C. Meanwhile, AVG and sodium thiosulfate (STS, an inhibitor of ethylene signaling) demonstrated the opposite results. This suggests that ethylene promotes lettuce seed germination under thermoinhibition, primarily by increasing endo-β-mannanase activity, which softens the hardness of the endosperm cell wall [31,36]. This is consistent with the findings that endo-β-mannanase activity in the endosperm of lettuce seeds at high temperatures may be regulated by ethylene [27]. In addition to its effect on the endosperm, ethylene may ensure that seeds absorb sufficient water during germination by maintaining a lower water potential in the embryonic region, thereby enabling proper germination [46]. An analysis of genetic, physiological, and gene expression data indicated that the mRNA abundance of the homolog of ethylene-signaling-related genes (*CTR1*) in lettuce generally decreased during seed-sucking at normal temperatures. However, the mRNA levels of heat-tolerant genotypes and heat-intolerant genotypes that sucked seeds at 35 °C remained high, and other genes related to ethylene signaling, *LsETR1*, showed a similar pattern, which, combined with the synergistic effect of ethylene and gibberellin, led to the further speculation that ethylene may induce the expression of *GA3ox1* through *LsERF1* and circumvent the inhibition of ABA through the action of GA, promoting lettuce seed germination at high temperatures (Figure 3). In addition, based on promoter motifs, *LsERF1* may promote the weakening of the endosperm cap and alleviate the level of thermoinhibition in lettuce seeds by directly stimulating the expression of *LsMAN1* [12]. In the life cycle of plants, the balance between ethylene and cytokinins is crucial for maintaining normal growth and development. However, this article focuses on the impact of thermoinhibition on lettuce seeds, limited to the four primary endogenous plant hormones. Based on current research findings, an increasing number of exogenous hormones and less abundant endogenous hormones have been shown to play a significant role in the thermoinhibition of lettuce seed germination. However, most studies have only examined the phenotypic and physiological aspects, with a lack of conclusions on the underlying genetic transcription and the interaction networks of these hormones. Further research and discussion are needed.

## 5. Inhibition of Physiological Metabolic Processes

Generally speaking, plants undergo complex physiological changes in response to stress conditions, and this aspect is also indispensable for studying thermoinhibition in lettuce seeds. There have been demonstrated that germination of Grand Rapids lettuce at 38 °C was significantly impaired, accompanied by a decline in ATP and total adenylate content, as well as adenosinergic charge within the seeds [88]. The researchers discovered that treating lettuce seeds with 100% oxygen and 10 mg/L agonist completely alleviated the thermoinhibition phenomenon. In contrast, the combination of oxygen and ethylene only partially alleviated it. Furthermore, it was discovered that the combination of carbon dioxide and ethylene completely overcame the thermoinhibition phenomenon in lettuce seeds at 35 °C. However, the effect of ethylene was essentially zero when carbon dioxide was absorbed by potassium hydroxide (KOH) [97]. It can be hypothesized that the effect of ethylene in the process of heat suppression in lettuce seeds is mainly dependent on the carbon dioxide concentration around the seeds rather than oxygen. Moreover, in air, AVG or NBD exhibited partial inhibition of seed germination, while the combination of the two agents exhibited severe inhibition of lettuce seed germination. However, oxygen plus agonist treatments almost completely alleviated the inhibitory effects they produced. Based on the experimental results, it is hypothesized that the effects of oxygen and agonists may have been to cause the seeds to avoid the ethylene requirements for germination or to increase the sensitivity of lettuce seeds to ethylene. Respiration can be divided into two categories: anaerobic and aerobic. At 38 °C, the alcoholic fermentation process was the dominant metabolic pathway in lettuce seeds. However, under the combination of oxygen and agonists, the seeds were allowed to undergo aerobic respiration. Consequently, the researchers concluded that the suppression of heat development in lettuce seeds is likely to be related to insufficient ATP content at high temperatures. Put another way, the reason why the combination of oxygen and agonist treatments slowed down the heat suppression may be related to the promotion of higher ATP content [88]. 

Intracellular ATP production in leaf lettuce seeds was severely inhibited when nitrogen was inhaled, while high concentrations of cyanide (CN) effectively inhibited seed germination and induced dormancy. However, at lower concentrations, cyanide promoted seed germination at both 25 °C and 15 °C [98]. The authors of the latter study postulated that the promotion of lettuce seed germination by low concentrations of cyanide at 25 °C may be due to a lowering of the energy threshold for germination below the level of dormancy induction. The processes involved in this phenomenon have not yet been fully elucidated. However, based on the feedback from the experimental data, it can be postulated that there is a correlation between changes in membrane potentials and the phenomenon under investigation. In conclusion, the effects of low levels of cyanide are analogous to those of gas and radiation in that they prevent the generation of dormancy, thereby promoting seed germination and embryonic growth [99,100]. Based on the above research findings, it is clear that changes in ATP content play a significant role in the thermoinhibition of lettuce seeds. Furthermore, ATP is closely related to physiological processes such as respiration and photosynthesis. Therefore, it is particularly important to study the genes and metabolites involved in these physiological processes behind the thermoinhibition of lettuce seeds, as well as their interactions with hormones.

## 6. Effects of Photosensitive Pigments

Photosensitive pigments play a pivotal role in the processes of seed germination and seedling de-yellowing [101]. In lettuce seeds, red light was found to elevate the upper temperature limit for seed germination, overcoming the thermoinhibition that occurs under dark culture. This indicates that the interaction between light and high temperature is likely to be mediated by the involvement of photosensitive pigments [45,49]. Specifically speaking, seeds of lettuce that matured at lower temperatures or under far-red light irradiation exhibited a maximum threshold for germination temperature than seeds grown at higher temperatures or under red light irradiation [21]. This was observed in more than one study [102,103]. Consequently, red light irradiation is capable of alleviating the thermoinhibition phenomenon of lettuce seeds within a certain temperature range for the majority of lettuce varieties. However, as the temperature increases, the response of the seeds to red light diminishes, resulting in thermoinhibition [104].

Among the five photosensitive pigments, phyB is the most important. Upon absorption of red light, phyB becomes more active and translocates to the nucleus, where it interacts with bHLH transcription factors in phytochrome interacting factors (PIFs) and promotes their degradation. phyB is not only able to sense light but also sensitive to temperature. As the temperature rises, phyB undergoes a transformation from Pfr to an inactive form, a process known as “thermal reversal”. This enables plant seedlings to quickly sense subtle changes in external temperature and to adjust their growth status in time to avoid the effects of heat stress [105]. Scientists have found that phyB is present in both the endosperm and the embryo, and the light-mediated inactivation of phyB accelerates the rate of ABA accumulation in the plant endosperm, preventing normal seed germination [106]. The determination of ABA and its metabolites revealed that the levels of both ABA and its catabolic metabolites were increased under red light treatment. Quantitative reverse transcription-polymerase chain reaction analysis showed that *LsNCED2* and *LsNCED4* were downregulated and *LsABA8ox4* was upregulated in lettuce seeds. It can be seen that ABA levels in lettuce seeds are directly regulated by photosensitive pigments, and this process is also related to the action of GA3. Finally, after red light irradiation, the ABA level in lettuce seeds decreased significantly more on the cotyledon side of the hypocotyl than on the hypocotyl side of the lettuce seeds. Since the cotyledon side of the hypocotyl is responsible for the seed germination process, the expression of genes related to the synthesis of ABA and GA, which are regulated by photosensitizing pigments, was concentrated in the hypocotyl, results echoing the conclusions of the previous section. These phenomena suggest that the regulation of ABA and other hormones by photochromes occurs at the light-sensitive site and regulates the thermoinhibition of lettuce seed germination at high temperatures [61].

## 7. Maternal Effect

Certain lettuce cultivars usually go into incipient dormancy after harvest as part of the natural developmental process of the seed, and the parent plant passes this mechanism on to its offspring, so the seeds must be stored to overcome the dormancy period [107]. Seed heat tolerance is a genetically controlled trait associated with the endosperm cascade [108]. Since the endosperm is a triploid tissue that develops mainly from maternal reproductive organs, heat tolerance is also influenced by maternal effects [109,110]. 

The heat tolerance of Greater Everglades lettuce, which is more tolerant to heat, and Veronica lettuce, which is more susceptible to disease, was measured by selecting for heat tolerance in Greater Everglades lettuce, as well as their orthologous and inverse hybrids. It was found that the F1 progeny of “Everglades” as the mother and “Veronica” as the father had a higher germination rate at 32 °C, while the F1 progeny of “Veronica” as the mother and “Veronica” as the father or the self-pollinated “Verônica” had a lower germination rate at 32 °C. This indicates that the heat tolerance of lettuce seeds is influenced by the genotype of the parent. In addition, at 32 °C, the experiment also discovered that the esterase content was higher in the F1 hybrid seeds of “Everglades” and “Everglades” and “Veronica”, while “Veronica” and “Everglades” had higher esterase content. “Everglades” and “Veronica” had high esterase content, while “Veronica” had the lowest esterase content in the F1 generation of hybrid seeds of “Everglades”. This indicates that lettuce seeds from heat-tolerant varieties have higher enzyme production than those from temperature-sensitive varieties, further confirmation of the relationship between heat tolerance and maternal effects in lettuce seeds [111]. 

Genetic control of heat tolerance in lettuce seeds can be attributed to one or a few genes, as shown in a randomized replicated experiment in which the heat-tolerant ‘Everglades’ was selected to be crossed with the heat-sensitive ‘Veronica’ to obtain seeds of F1, F2, and F2:F3 generations. Although additive effects were more significant than non-additive effects, the narrow-sense heritability of heat-tolerance and inhibitory traits in lettuce seeds was high, and their early selection was good enough to predict and select for genetic gain [111]. In summary, the strategy of producing heat-tolerant lettuce offspring through hybridization between different lettuce varieties is feasible. However, the hybridization process heavily relies on the purity of the parents and the influence of external environmental factors. Therefore, whether it is possible to design and optimize other genetic methods to produce heat-tolerant offspring remains an open question. 

## 8. Genetic Expression

The phenomenon of thermoinhibition in lettuce seeds under a high-temperature environment is an important topic in plant physiology research, and the current literature mainly focuses on how heat stress affects the physiological functions of seeds, which includes the changes of phytohormones [112], the activity of photosensitive pigments [101,102,103,104,105,106,107], the inhibition of physiological metabolism [97,98,99,100], and the maternal effect [113]. With the development of science and technology, especially the continuous advancement of sequencing technology, researchers have begun to explore in depth the changes in gene expression and metabolites associated with heat stress in lettuce seeds. Recent studies have shown that the phenomenon of seed thermoinhibition is the result of a combination of factors. For example, the authors of [111] found that in heat-sensitive lettuce genotypes, the seed thermoinhibition phenomenon at high temperatures of 35 °C could be significantly eliminated by knocking out or silencing the *LsDOG1* gene. This finding emphasized the importance of the *LsDOG1* gene in the thermal response of seeds. It was also observed that overexpression of the *DOG1* gene in Arabidopsis and lettuce seeds reduced the heat tolerance of the seeds. These results suggest that the expression level of the *DOG1* gene is extremely sensitive to changes in external temperature and plays a key role in seed development and suckering [114]. Therefore, future studies need to further explore the specific mechanism of action of the *DOG1* gene and other related genes in seed heat response. 

Numerous researchers have used an untargeted metabolomics approach to investigate metabolite changes in lettuce seeds under heat stress. The results showed that there are differences in metabolic coping strategies between heat-sensitive and heat-tolerant lettuce seeds at high temperatures. At 21 °C, heat-tolerant lettuce seeds accumulated more organic acids, amino acids, sugars, sterols, phenolic compounds, and terpenoids, whereas, at 35 °C, heat-tolerant lettuce seeds produced more amino acids, organic acids, sugars, sesquiterpene lactones, sterols, and fatty acid derivatives [80]. Transcriptomic and metabolomic analyses of two heat-sensitive and two heat-tolerant lettuce cultivars identified a total of 15 different metabolites, including three amino acids, one carbohydrate, eight phenolic compounds, one terpene, and one lignan. The transcriptome data showed that 1449 genes (C2 vs. H2), 810 genes (C3 vs. H3), 557 genes (C4 vs. H4), and 1333 genes (C6 vs. H6) were initially screened. Finally, the combined metabolomic and transcriptomic analysis revealed that a large number of genes and metabolites were involved in the production of flavonoid substances during the thermal response. Among the 31 reported transcription factors, 25 showed different degrees of upregulation, A few of the most notable genes are *MYB*, *WRKY*, *NACS*, *bHLHs*, *MADS*, *AP2/ERF*, and *Bzip* [22,78,115]. 

As indispensable molecules in living organisms, proteins have a variety of important functions in life activities. In recent years, with the development of molecular biology techniques, researchers have begun to explore the role of proteins in the response of plants to environmental stress, especially high-temperature stress. Such as, when lettuce seeds were tested for germination under high-temperature conditions, the protein content of both germinated and ungerminated seeds showed a decreasing trend. This finding revealed the importance of proteins in the seed germination process and showed that the accumulation of 12 specific proteins and their mRNAs was positively correlated with the germination ability of seeds. Through annotation analysis, the researchers further found that these proteins are involved in a variety of biological processes, including methionine metabolism, ethylene production, lipid conversion, cell elongation, glycolysis, isoprenoid biosynthesis, and aldehyde detoxification. The proper functioning of these processes is essential for seed germination, and interference with the phenomenon of thermoinhibition can lead to blockage of these processes, which in turn prevents normal seed germination [111]. 

In addition, studies of the effects of protein accumulation and changes in gene expression on seed germination have also shown that the mevalonate (MVA) pathway plays a role in seed germination and thermoinhibition, particularly in the biosynthesis of isoprenoids. Cytokinins produced by the MVA pathway have a positive effect on lettuce seed germination. The MVA pathway also produces other bioactive substances such as farnesyl diphosphate and geranyl diphosphate, which were also detected during seed germination. Concurrently, the intricacy of the intrinsic mechanism of action of the MVA pathway is augmented by the multitude of close interactions between the MVA pathway and the MEP pathway [116].

From the above discussion, it is clear that the role of proteins during seed germination and their response mechanisms under high-temperature stress are important parts of lettuce adaptation to adversity, and in the future, it will be necessary to further reveal the specific mechanisms of action of these proteins and metabolic pathways, as well as how to enhance lettuce adaptation to high-temperature adversity through genetic modification or environmental regulation.

## 9. Other Factors

In the field of lettuce seed heat stress research, in addition to the aspects where intensive progress has been made, there are some new findings and results that deserve attention. For example, the application of nanotechnology in seed heat-stress research is gradually gaining attention. Since carbon nanomaterials have not been studied as much as other abiotic stresses in the direction of thermoinhibition of seeds, the specific mechanism of action of alleviating thermoinhibition by carbon nanotube (CNP) pretreatment still needs to be further explored; however, according to existing studies, nanomaterials are able to regulate the expression of heat shock proteins and antioxidant functions, and these may be of significant effect in improving the heat tolerance of seeds [117,118]. 

The potential of nitrate, as an indispensable nitrogen source for plant growth, in alleviating heat stress in lettuce seeds cannot be ignored [26]. Experimental results showed that Pioneer 1 lettuce seeds germinated best under certain temperature conditions, namely, 11–19 °C under light conditions and 13–15 °C under dark conditions. It was also found that water vapor produced by nitric nitrogen, Fe(III)CN, and acidified nitrite was effective in alleviating the thermoinhibition phenomenon of seeds under light conditions. Meanwhile, the inhibitory effects of SNP, Fe(III)CN, nitrite, and nitrate must depend on the involvement of nitric oxide (NO) [26]. 

In addition to these findings, studies have demonstrated that with the implementation of appropriate pretreatment measures, such as the hydration of lettuce seeds prior to sowing, whether with water, a salt solution, or a moist solid carrier, can facilitate the acquisition of varying degrees of high-temperature tolerance in lettuce and other vegetable seeds, thereby enabling successful germination at high temperatures [119]. Moreover, soaking ‘Minetto’ lettuce seeds in 1% K_3_PO_4_ for 20 h at 15 °C was found to alleviate thermoinhibition to a greater extent than after soaking in water [120]. Additionally, it was demonstrated that seeds of the ‘Mesa 659’ lettuce variety exhibited a germination rate of greater than 80% at 35 °C following an initial soaking period on moist Micro-Cel E, a synthetic calcium silicate [121]. 

In addition, an increasing number of studies have found that the germination of lettuce seeds at high temperatures is influenced by a variety of physiological and metabolic processes, including but not limited to methionine metabolism, lipid transformation, aldehyde detoxification, and cell expansion. These processes are closely related to the occurrence of thermoinhibition in lettuce seed germination, and, therefore, they may be one of the key factors contributing to the phenomenon of thermoinhibition. [122]. 

## 10. Conclusions

In agricultural crop cultivation, the synchronization and rapidity of seed germination are crucial factors for improving crop yield and quality. However, the problem of dormancy can affect the normal germination of seeds. In general, seed dormancy is defined as a condition in which a surviving dormant seed fails to germinate under conditions suitable for germination [123]. Conversely, thermoinhibition occurs when non-dormant seeds fail to germinate under high-temperature adversity. This phenomenon is often confused with dormancy due to the similarity in the mode of regulation to which both are subjected [51]. It is crucial to clarify the physiological and genetic differences between the two, and despite the large amount of research in this area, there is still a need for further explanation and demonstration of many key aspects. With the continuous development of molecular biology and omics technologies, it is expected that the application of seed dormancy/germination omics (dormomics/germinomics) techniques will help to elucidate the mechanisms underlying seed thermoinhibition of germination.

In contrast, lettuce is a typical crop for thermoinhibition and is an excellent material for studying the phenomenon of thermoinhibition. In this paper, several dimensions of current research on the thermoinhibition of lettuce seed germination that are the most concentrated on are elaborated, including the effects of embryo structure, changes in seed physiological functions, gene expression, protein accumulation, and interactions between transcription and metabolites. Among these, the effects of endosperm hardening and the interactions between ABA and hormones such as GA and ETH have been studied more in each crop. However, the complex interactions between these different levels have not been fully elucidated in lettuce. This is because these mechanisms and processes have not been fully investigated and it is beyond the scope of this paper to explore all these interaction mechanisms. It is important to bear in mind the following point, in this paper, we have only made a preliminary reference and summarized some of the genes and transcription factors related to the thermoinhibition phenomenon in the literature, while an in-depth understanding of the thermoinhibition phenomenon will help workers to have a more comprehensive understanding of the seed germination process, which requires workers to further explore the association between genetic factors and transcription and metabolism genes related to the process of thermoinhibition in lettuce seeds. At the same time, in order to save readers’ time and supplement the existing research results, we have summarized some experimental results of different substances or treatments on the germination and growth of lettuce seeds under thermoinhibition and placed them in Table 2 for reference.

## 11. Future Perspectives

As the challenges of global climate change become increasingly severe, it is urgent to study the phenomenon of thermoinhibition in seeds of various plants. Research on this topic is abundant in plants such as Arabidopsis and tomato, but relatively scarce in lettuce. Therefore, drawing on the practices of these plants regarding thermoinhibition is very meaningful for lettuce researchers. Hormones have very complex interactions with each other, and temperature can directly affect various processes in plant hormone pathways. Although there is a lot of research on hormones in thermoinhibition, significant knowledge gaps and challenges still exist, which provide opportunities for future investigation and research. For example, in addition to the study of the traditional five major plant hormones, research on the effects of newer exogenous hormones such as strigolactones, karrikins, and salicylic acid on the thermoinhibition of seed germination in different plants is gradually increasing. Studies have found that the phenomenon of thermoinhibition can reveal the clear role of strigolactones in promoting seed germination in Arabidopsis. Both strigolactone biosynthetic and signaling mutants have increased sensitivity to thermoinhibition in Arabidopsis seeds. The synthetic strigolactone GR24 can effectively restore the germination of heat-inhibited biosynthetic mutant seeds and it was ultimately found that strigolactones alleviate thermoinhibition by modulating the levels of GA and ABA [9]. Not just that, through experiments, it was found that maize seedlings pretreated with salicylic acid (SA) showed improved heat and cold tolerance. Among them, maize seedlings pretreated with 300 mmol·L^−1^ SA had the greatest improvement in heat tolerance after being subjected to 46 °C high-temperature stress for 2 days [139]. Our research group also found that after treating the temperature-sensitive lettuce variety Qisegreen with 0.2 μmol·L^−1^ karrikin, both in soil conditions and in petri dish environments, compared with untreated and water-primed controls, the karrikin treatment group exhibited significantly enhanced germination rate. Therefore, future research on thermoinhibition of lettuce seed germination can appropriately focus on the mechanisms and effects of some hormones or other physiological secondary metabolites that have not yet been thoroughly studied, and associate and extrapolate from existing research results.

In recent years, contemporary methods including genome sequencing, transcriptomics, metabolomics, and proteomics have been employed to understand the complex responses of seeds, such as lettuce, to heat stress. Additionally, some key genes associated with lettuce seed thermoinhibition have been preliminarily identified. A further study also emphasized that multi-omics approaches can effectively elucidate the “entire set of regulatory components in a cell, including regulatory elements, genes, mRNAs, proteins, and metabolites”, as well as the responses of various plants to temperature stress [140]. For instance, through transcriptome analysis, it is indicated that genes involved in photosynthesis, redox reactions, and auxin activity are upregulated under heat treatment [141]. Through untargeted metabolomics analysis, it was found that heat-sensitive and heat-tolerant varieties of seeds adopt different metabolic strategies during germination under high-temperature stress; the total amount of organic acids, amino acids, sugars, sterols, phenolic compounds, and terpenoids is higher in heat-sensitive seeds than in heat-tolerant seeds [22]. Proteomics has also been used to study seed stress germination in many species, such as Arabidopsis [142], rice [143], and pea [144], focusing on responses to normal conditions or various chemical treatments, such as GA [145], ABA [145], and salicylic acid [146], as well as environmental stresses, such as salinity [147,148]. However, the mechanisms regulating lettuce seed germination and thermoinhibition are far from fully understood, with most results being preliminary based on single-omics studies, especially with fewer results from integrated multi-omics analyses. Therefore, there is good reason to believe that this will be a hot research field and direction in the future. 

Finally, with the rapid development of artificial intelligence, how machine learning can be effectively used to identify seed viability and intrinsic chemical composition changes has become a hot topic, and many scholars and laboratories have achieved good results. For example, researchers attempted to use neural networks to distinguish lettuce varieties through Kohonen’s self-organizing map and organize them according to their tolerance to seed thermoinhibition. In the end, the discriminant analysis identified Everglades and Luiza as heat-tolerant lettuce varieties [149]. In addition, another laboratory utilized a self-built near-infrared spectral detection system to extract the spectra of single tomato seeds in the range of 980~1700 nm and established qualitative analysis models for tomato seed heat damage using partial least squares discriminant analysis (PLS-DA) and support vector machines (SVM). The final experimental results showed that the total accuracy of the two discrimination models on the validation set was greater than 96%, and both could be effectively used for the discrimination of tomato heat-damaged seeds [150]. Additionally, researchers used a self-built diffuse reflectance near-infrared spectral acquisition system to establish quantitative prediction models for protein, oil content, and starch in corn seeds, with correlation coefficients ranging from 0.66 to 0.89, providing a new method for the determination of seed chemical components [151]. Based on the current research results on machine learning, the focus is mainly on seed viability identification and the prediction of intrinsic physiological substance content, which can reduce human error and provide precise quantification. Therefore, if used to study the underlying mechanisms and effects behind lettuce seed germination thermoinhibition, it is best to combine machine learning with the methods mentioned earlier, using interdisciplinary cross-research to address related issues. This analytical approach will be the main theme of future scientific research. 

## Figures and Tables

**Table 1:** Some genes known or predictive to affect the thermoinhibition of Lettuce seeds. The Lactuca sativa gene names are used; LOF loss of function, ND not determined.

Gene	Biological Function	Thermoinhibition Phenotype of LOF Mutant	References
ABA biosynthesis and signaling			
*LsABA1/ZEP*	ABA biosynthesis	Caused by single-base mutations, Resistance to thermoinhibition	[42]
*LsABI3*	ABA signaling and Seed maturation	Resistance to thermoinhibition	[49,65]
*LsABI5*	ABA signaling	Resistance to thermoinhibition	[66]
*LsNCED4*	ABA biosynthesis	Resistance to thermoinhibition	[67]
*LsABA8ox4*	ABA biosynthesis	The accumulation of ABA is blocked, Resistance to thermoinhibition	[49,68]
*LsSDR1*	ABA biosynthesis	inhibit ABA synthesis, Resistance to thermoinhibition	[60]
*LsNCED2* *LsNCED3*	ABA biosynthesis	ND	[6,69,70]
Ethylene biosynthesis and signaling			
*LsACS1* *LsACO2*	Ethylene biosynthesis	Resistance to thermoinhibition	[49,71]
*LsERF1*	Ethylene signaling	Sensitive to thermoinhibition	[12]
*LsCTR1*	Ethylene signaling	Resistance to thermoinhibition	[49]
Gibberellin biosynthesis and signaling			
*LsGA3ox1*	Gibberellin biosynthesis	Sensitive to thermoinhibition	[6,61,72]
*LsGA2ox1*	Gibberellin biosynthesis	Resistance to thermoinhibition	[73]
*LsCPS1*	Gibberellin biosynthesis	ND	[49]
*LsKS1*	Gibberellin biosynthesis	ND	[49]
*KO1*	Gibberellin biosynthesis	ND	[49]
*LsRGL2*	GA signal suppression factor	Resistance to thermoinhibition	[74,75,76]
Other			
*LsDOG1*	Induction and maintenance of seed dormancy	Resistance to thermoinhibition	[51,77,78]
*LsHSP70*	Mini-heat shock proteins that are responsive to thermostress	forecast More sensitive to thermoinhibition	[79]
*LsC4H* *Ls4CL*	Flavonoids synthesis	forecast More sensitive to thermoinhibition	[80]
*LsF3H* *Ls3GT*	Encoding Glycosyltransferase	High expression enhances the antioxidant activity of lettuce under heat stress and inhibits the formation of free radicals	[80]
*LsWRKY33*	Regulating gene expression related to heat response	forecast More sensitive to thermoinhibition	[81]
*LsCBF*	Regulating the response of lettuce to abiotic stress	ND	[22,82]
*Ls ERF085* *Ls ERF116*	Regulating complex stress response pathways	ND	[83]
*LsMAN1*	Encoding inlier β—Mannanase	forecast More sensitive to thermoinhibition	[33]
*LsSOC1* *LsMAPK4* *Ls GASAs*	Regulatory factors for lettuce bolting under high temperature	Resistance to thermoinhibition, Delayed bolting and flowering	[84,85,86]

**Table 2:** Effects of Different Priming Methods on Germination and Growth of Lettuce Seeds under High-Temperature Thermoinhibition.

Reagents and Materials	Treatment Method	Optimal Concentration	Lettuce Variety	Effect under High Temperature Post-Treatment	References
Melatonin	Foliar Spray	50 μMol/L	Heat-sensitive variety: Shui San No. 3	Effectively reduces membrane permeability of lettuce seedlings, enhances antioxidant system activity, and alleviates thermoinhibition	[124]
Spermidine	Foliar Spray	1 mMol/L	Heat-tolerant: G-S59 Heat-sensitive: P-S11	Effectively reduces seedling membrane permeability and malondialdehyde content, alleviating thermoinhibition	[125]
Silk Protein	Seed Soaking	4 h, 50 mg/L (large molecular weight) and 100 mg/L (small molecular weight)	Rabbit, Emperor, Santa, Jinke	Germination rate, germination potential, vigor index, and fresh weight per plant of all four varieties are significantly higher than the control	[126]
AM Fungi	Inoculation	100 mL Mycorrhizal Fungi	Heat-tolerant:Double Head Ball Lettuce	Significantly improves heat tolerance of lettuce at 35 °C, maintains the integrity of the electron transport chain on the acceptor side of the PSII system in seedling leaves, and enhances photosynthetic capacity	[127]
BR/PEG	Seed Soaking	10%PEG	No variety information	BR severely inhibits lettuce germination under high temperatures, and the appropriate concentration of PEG significantly improves lettuce germination rate and uniformity	[128]
PEG	Priming	25%PEG	Daguo 659 Coated Seeds	Significantly improves the germination rate and germination potential, promotes seedling height, fresh weight, and dry weight of coated lettuce seeds	[129]
PEG + Fungicide	Priming	−1.25 Mpa PEG + Funaben T/Apron 35 SD	Krolowa	Both significantly reduce fungal infection of seeds, improve seed germination rate and germination index, and alleviate seed thermoinhibition	[130]
Vermiculite	Priming	Seed Dry Weight:Vermiculite Dry Weight:Water = 5:10:9	Heat-tolerant Erbaipi Green Qing	After priming, lettuce seed germination potential, germination rate, seedling dry and fresh weight, SOD, POD, and other antioxidant enzyme activities are higher than the control	[131]
GA3 + 6-BA	Priming	5 mg/L	Glass Lettuce Purple Leaf Lettuce	All germination indicators and antioxidant enzymes are significantly improved compared to the control group, increasing seedling chlorophyll content, improving respiratory metabolism levels, and increasing seedling Hsp70mRNA content	[132]
PEG	Priming	−13.00 Mpa 7 d	Italian Lettuce	Significantly improves seed germination potential, germination rate, antioxidant enzyme activity, and germination time	[133]
KH_2_PO_4_	Priming	−1.5 Mpa KH_2_PO_4_ + 10 μM ACC 20 h Dark Conditions	No variety information	Final germination rate, germination synchronicity, and emergence rate are significantly improved	[134]
Water	Priming after Sowing	55% Moisture Content	Patriot	Emergence rate under high temperatures is significantly improved compared to the control group	[135]
PGR (Plant Growth Regulator)	Drum Perfusion	8 °C 3 d 1 mg/L^−1^GA_3_ + 1 mg/L^−1^BA + 390 mg/L^−1^ETH	Heat-sensitive:Hua Hong	Weakens the cytoplasmic condensation and microendosperm structure of the endosperm cells under high temperatures, increases the production of metabolic enzymes, and promotes seed germination	[136]
Red Light, Far-Red Light	Irradiation after Hydration	Red Light 24 h, Far-Red Light 216 h	Tango cutting lettuce, Ideal Cos lettuce, Gallega de Invierno, and Milanesa butterhead lettuce	Red light treatment at 25 °C improved the germination of lettuce seeds under dark conditions; both red and far-red light can prolong the lifespan of lettuce seeds	[137]
GA_3_	Priming	100 mg/L 15 °C 18 h Dark Conditions	Great Speed Lettuce Shooter 101	Germination rate, germination potential, germination index, vigor index, seedling fresh and dry weight, and antioxidant enzyme activity of the two lettuce varieties are significantly increased	[138]
